# In vivo exploration of synaptic projections in frontotemporal dementia

**DOI:** 10.1038/s41598-021-95499-1

**Published:** 2021-08-09

**Authors:** Eric Salmon, Mohamed Ali Bahri, Alain Plenevaux, Guillaume Becker, Alain Seret, Emma Delhaye, Christian Degueldre, Evelyne Balteau, Christian Lemaire, André Luxen, Christine Bastin

**Affiliations:** GIGA Cyclotron Research Centre, University of Liège, B30 Sart Tilman, 4000 Liège, Belgium

**Keywords:** Neuroscience, Biomarkers, Diseases, Neurology, Pathogenesis, Signs and symptoms

## Abstract

The purpose of this exploratory research is to provide data on synaptopathy in the behavioral variant of frontotemporal dementia (bvFTD). Twelve patients with probable bvFTD were compared to 12 control participants and 12 patients with Alzheimer’s disease (AD). Loss of synaptic projections was assessed with [^18^F]UCBH-PET. Total distribution volume was obtained with Logan method using carotid artery derived input function. Neuroimages were analyzed with SPM12. Verbal fluency, episodic memory and awareness of cognitive impairment were equally impaired in patients groups. Compared to controls, [^18^F]UCBH uptake tended to decrease in the right anterior parahippocampal gyrus of bvFTD patients. Loss of synaptic projections was observed in the right hippocampus of AD participants, but there was no significant difference in [^18^F]UCBH brain uptake between patients groups. Anosognosia for clinical disorder was correlated with synaptic density in the caudate nucleus and the anteromedial prefrontal cortex. This study suggests that synaptopathy in bvFTD targets the temporal social brain and self-referential processes.

## Introduction

Frontotemporal lobar degeneration (FTLD) is a heterogeneous group of diseases that affect anterior brain regions associated with personality, behavior, executive functions and language deficits. The pathophysiology in most cases of FTLD includes an abnormal intracellular accumulation of disease specific proteins, such as tau or transactive response-DNA binding protein-43 (TDP-43). FTLD is associated with ventromedial and dorsolateral frontal and anterior temporal gray matter loss and glucose hypometabolism^1^. Even patients with very mild dementia may show atrophy in anterior cortical (comprising paralimbic frontal) and subcortical (striatal and thalamic) regions^2^. There is also a significant white matter degradation in association and commissural tracts (such as the uncinate and longitudinal fasciculi and the corpus callosum) in FTLD^3^. Using fMRI, the intrinsic connectivity network disrupted in the behavioural variant of frontotemporal dementia (bvFTD, a subgroup of FTLD, was shown to involve the anterior cingulate cortex, frontal insula and striatum, related to emotional salience processing capacities^4^.

Accumulating evidence indicates that neuronal death in neurodegenerative diseases is preceded by disruption of synapses. Abnormal brain connectivity (connectopathy) or synaptopathy have been described in FTLD^5–7^. Extensive synaptic loss and reduction in the number of spines have been documented post-mortem in the diseased cortex^8–10^. More precisely, a significant decrease in synaptic density measured with synaptophysin was reported in the superficial layers of the prefrontal cortex of FTLD cases compared with normal controls in some^10^, but not all studies^11^. In Pick’s disease, synaptophysin immunoreactivity was also reduced in the outer molecular layer of the hippocampal dentate gyrus^12^.

The aim of this study was to measure brain synaptic density in vivo using synaptic vesicle protein 2A-PET in patients with bvFTD^13–15^. SV2A-PET has been recently used to assess synaptopathy in different neuropsychiatric conditions^13–15^, and frontotemporal involvement was recently reported in a patient with C9orf72 mutation^16^. We anticipated loss of synaptic density in the frontal and temporal poles and we searched for precise regional loss of synaptic projections. We have a particular interest in anosognosia as an early marker of neurocognitive disorders^17^, and we anticipated that decreased awareness for clinical symptoms, a frequent behavioral disorder observed in about 85% of bvFTD patients^18^ would be related to frontal and/or temporal synaptopathy^19^.

## Results

### Neuropsychological assessments

All values for cognitive performance and anosognosia measures in bvFTD and AD patients were significantly different from those in controls (Table 1). There was no significant difference between AD and bvFTD groups.

**Table 1:** Demographic and clinical data.

	bvFTD	AD	Controls
Age (years)	73.5 ± 7.6	74.1 ± 8.6	71.4 ± 5.2
Sex (W/M)	4/8	5/7	5/7
Education (years)	11.5 ± 4.1	11.8 ± 3.7	13.1 ± 3.6
Disease duration (years)	5.2 ± 3.4	6.2 ± 3.9	N/A
MMSE	25.4 ± 3.1	25.1 ± 2.6	29.1 ± 1.1
CDR (0.5/1/2)	1/8/3	2/10/0	N/A
DMS48 (% correct)	86.9 ± 14.9	85.9 ± 8.8	98.2 ± 2.1
Fluency (letter p)	13.3 ± 4.4	17.9 ± 7.7	20.4 ± 5.2
AQ-D difference	15.1 ± 13.3	5.4 ± 14.2	1.9 ± 5.9
MARS discrepancy score	0.4 ± 0.4	0.1 ± 0.4	0.0 ± 0.1

*W* women, *M* men, *MMSE* mini mental state exam, *CDR* clinical dementia rating, *AQ-D* Anosognosia Questionnaire for Dementia, *MARS* memory awareness rating scale.

### SV2A comparisons

**Figure 1:**
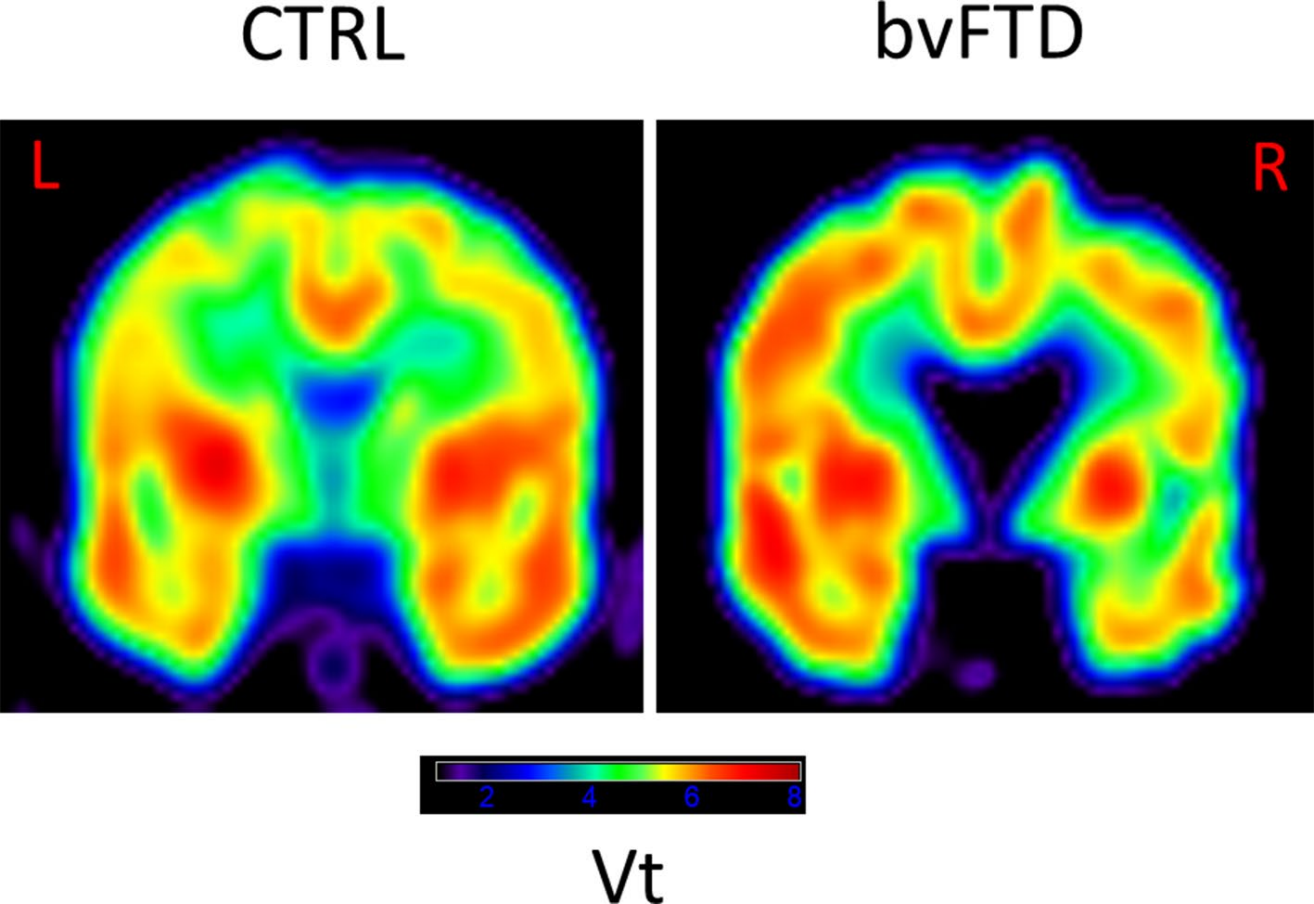
Coronal PET images of the brain distribution volume (Vt) of [^18^F]UCBH in a bvFTD patient and a control participant. [color required].

**Figure 2:**
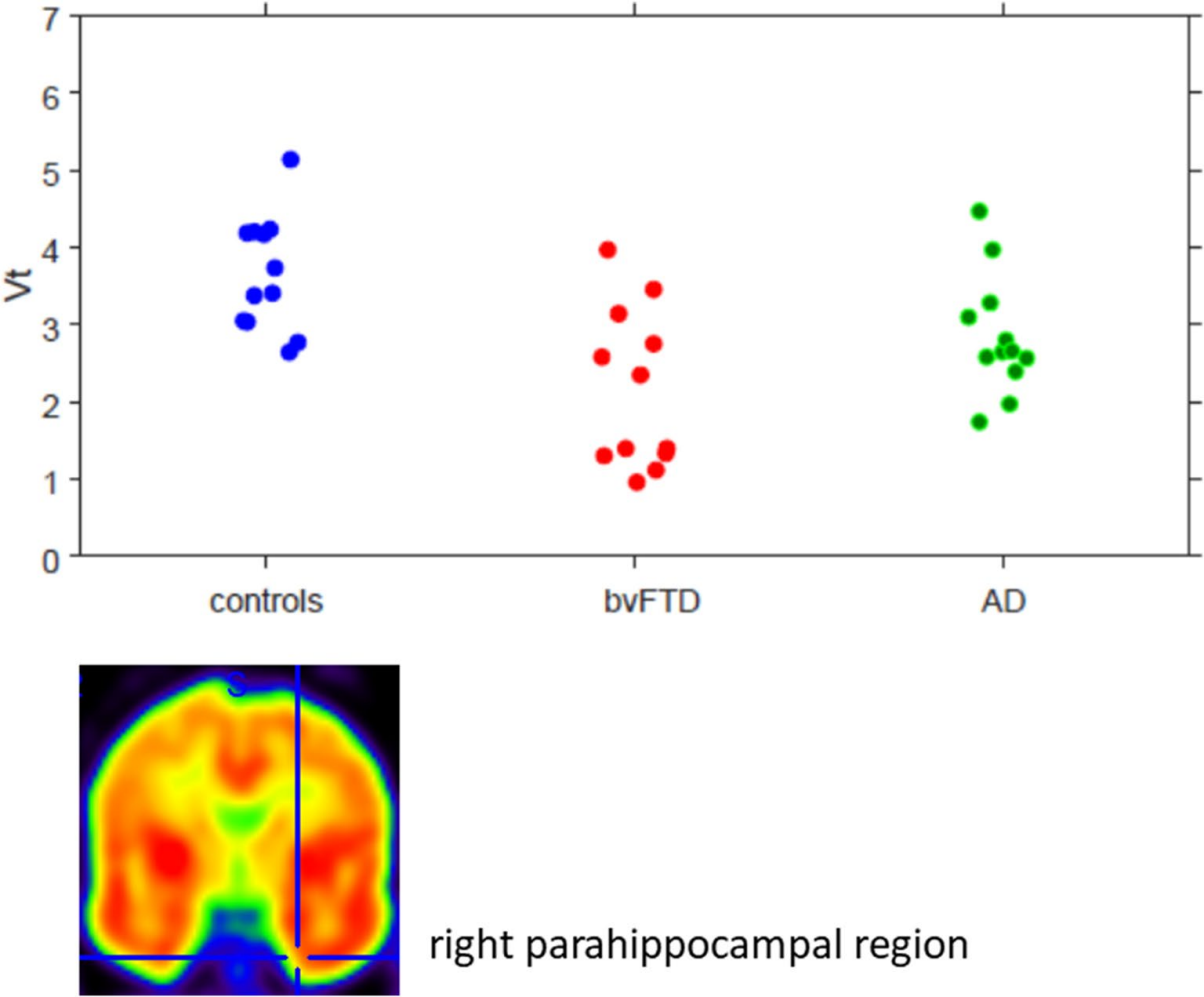
Plots of [^18^F]UCBH distribution volumes (Vt) measured in the right parahippocampal cortex (see cross on the figure) of control participants and patients with bvFTD and AD.

The brain distribution of [^18^F]UCB-H is illustrated in Fig. 1. For the bvFTD sample, there was a trend (P_FWE_ corr = 0.077) for a decrease in the distribution volume of [^18^F]UCB-H in a right parahippocampal region (BA36), close to the amygdala (Fig. 2 and Table 2). Loss of Vt in the cluster, calculated as (mean patients − mean controls)/mean controls × 100, was 41% compared to the control value.

**Table 2:** Neuroimaging results.

	x	y	z	Voxels	Z
**Comparison bvFTD < CTRL**					
R. Parahippocampal gyrus	18	− 2	− 38	85	4.03
**Comparison AD < CTRL**					
R. Hippocampus	14	− 8	− 18	80	4.06
**Correlation AQ-D difference in bvFTD**					
R. Caudate	12	16	6	574	4.52
m. AMPFC	4	58	8	492	4.26
**Correlation AQ-D difference in AD**					
R. Posterior hippocampus	12	− 42	8	431	4.10
**Atrophy in bvFTD < CTRL**					
m. AMPFC	− 8	68	− 2	300	3.84
**Atrophy in AD < CTRL**					
L. Parietal	− 62	− 62	30	1146	5.10
R. Posterior hippocampus	36	− 32	− 10	787	5.06

*AQ-D* Anosognosia Questionnaire for Dementia, *AMPFC* anteromedial prefrontal cortex, *R* right, *L* left, *m* medial.

We ran a seed to voxel connectivity analysis using CONN toolbox implemented in MATLAB^20^ and resting-state fMRI from 27 healthy elderly volunteers from an independent sample (Supplemental Table 1), and the right parahippocampal seed, corresponding to the peak of decreased synaptic projections in the bvFTD group (Supplemental Fig. 1). It was largely connected to the bilateral temporal poles and the right orbitofrontal cortex (p < 0.05 False Discovery Rate-corrected at the voxel level).

For the AD sample, there was a trend (P_FWE_ corr = 0.078) for a reduced synaptic density in the right hippocampus. Loss of Vt in the cluster was 33% compared to controls.

When patient groups were directly compared, there was a non-significant (but p < 0.001, uncorrected) decrease in Vt in the left precuneus (BA7) in AD compared to bvFTD.

### SV2A correlations

**Figure 3:**
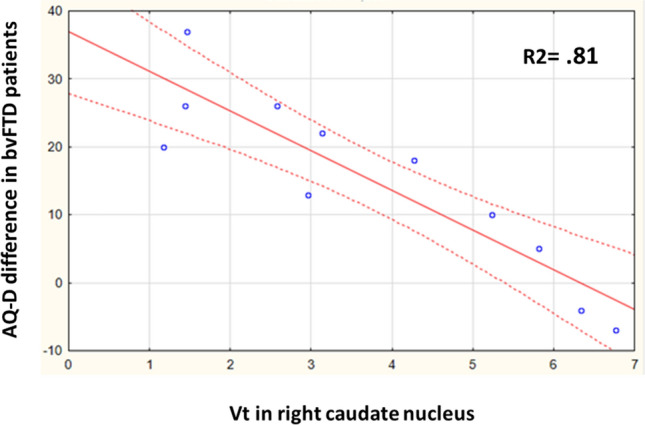
Graphic representation of the correlation between [^18^F]UCBH distribution volumes (Vt) in caudate nucleus and anosognosia for clinical symptoms in bvFTD.

For the bvFTD group, there was a significant negative correlation between anosognosia measured with the AQ-D and Vt in the right caudate head (r = − 0.89, pFWE = 0.023, Table 2 and Fig. 3) and a trend with Vt in the right frontal pole (r = − 0.87, pFWE = 0.058). The latter correlation was significant (pFWE = 0.009) when compared to controls or to AD participants.

We ran seed to voxel connectivity analysis using CONN toolbox and fMRI from our 27 healthy elderly volunteers, and caudate nucleus was connected with a large cluster centered on the anterior cingulate cortex, comprising the frontal pole (p < 0.05 FDR-corrected at the voxel level, Supplemental Fig. 2).

For the AD group, there was a trend for a negative correlation between anosognosia measured with the AQ-D and the left posterior hippocampus (pFWE = 0.088) when compared to controls.

### VBM analyses

For the bvFTD group compared to controls, there was a trend for atrophy in the medial prefrontal cortex (p < 0.001 uncorrected at the voxel level and pFWE = 0.032 at the cluster level, Table 2).

For the AD group compared to control, there was an atrophy of the right posterior hippocampus (pFWE = 0.010) and the bilateral posterior associative cortices (pFWE = 0.036, Table 2).

The direct comparison between patient groups did not provide any significant difference. There was no significant correlation with the clinical data.

**Figure 4:**
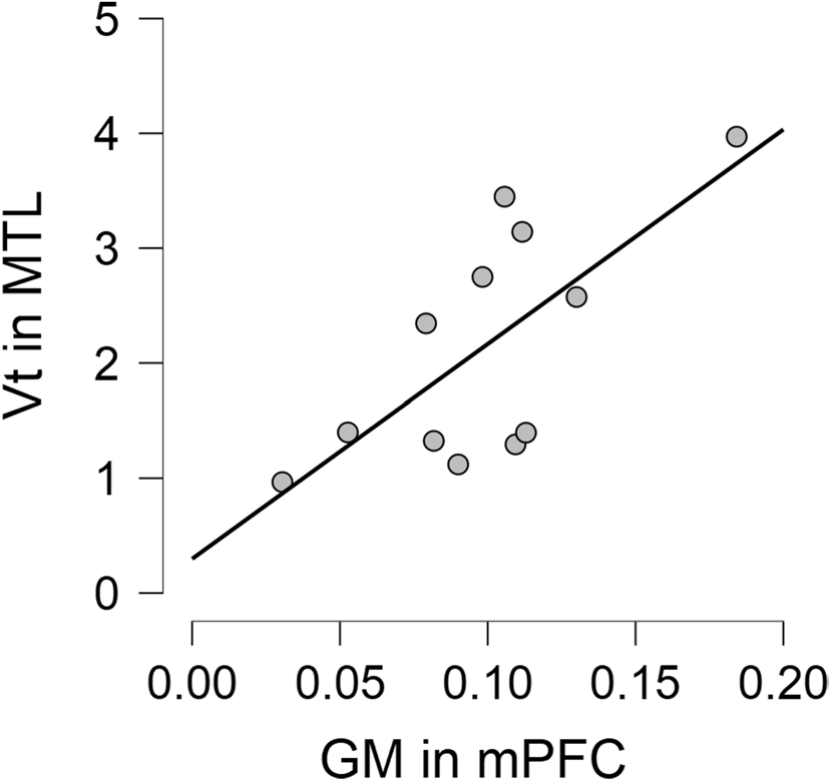
Correlation between GM density in medial prefrontal cortex and synaptic loss in medial temporal cortex.

We computed a correlational analysis between frontal atrophy (GM density corrected for individual brain sizes) and medial temporal synaptic loss to properly show the distribution of individual values for both neuroimaging data (Fig. 4). The correlation is significant (R = 0.70 and p = 0.010).

## Discussion

This exploratory study showed a trend for synaptic loss measured in vivo with SV2A-PET in the anterior parahippocampal gyrus of our sample of bvFTD patients, while atrophy was predominant in the ventromedial prefrontal cortex. The data in the AD sample were previously reported in a larger population^21^. Since comparison with bvFTD patients did not provide any significant differences, the results in AD will not be further discussed.

The anterior parahippocampal region corresponds to the rostral perirhinal cortex, that shows high probabilities of connection with the temporal pole, the orbital frontal regions and the frontal pole in the literature^22^ and in our connectivity analysis of fMRI in an independent sample of healthy elderly volunteers. The uncinate fasciculus is the connecting tract between those regions^23^ and its involvement in bvFTD would be related to loss of synaptic projections to the medial temporal lobe^3^. The right parahippocampal region (BA36), close to the amygdala, is part of the «social brain», involved in perception of socially salient stimuli^24^ and of the limbic network^25^. A relationship between atrophy in the perceptual social network and lack of attention to social cues was previously reported in bvFTD^24^.

Principal component analysis of FDG-PET previously showed that both frontal and temporal involvement are important in bvFTD^26^. The importance of temporal involvement in bvFTD was recently re-emphasized with TDP-43 studies^27^. TDP-43 pathology in bvFTD would be initiated in the orbitofrontal cortex and amygdala, progressing then to frontal and temporal cortices before affecting the motor system, visual cortex and cerebellum^28^. Patients with bvFTD are associated with TDP-43 type A, involving the frontal, temporal and parietal lobes and type B, involving the hippocampus^29^. The temporal lobes are shown to be critically affected in C9orf72 patients linked to TDP-43 pathology types A and B^30^, while medial temporal structures are less involved in GRN mutations^28^. Screening for C9orf72 abnormality was accepted by three patients in our sample but it was negative. Temporal involvement was also demonstrated in bvFTD with tau pathology^31,32^.

**Figure 5:**
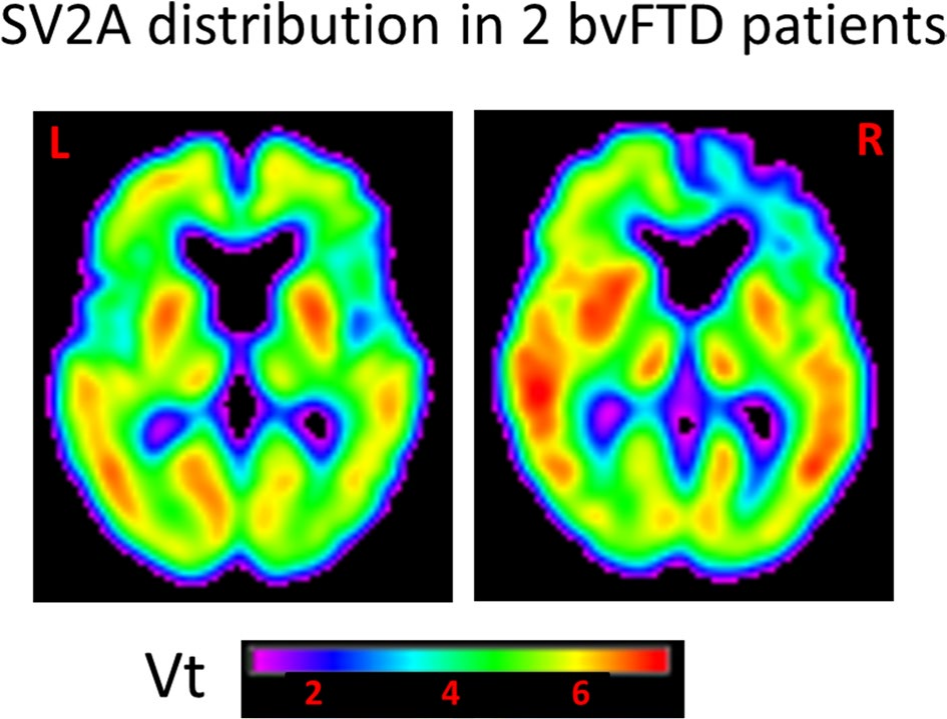
Variable frontal [^18^F]UCBH distribution (Vt) in bvFTD patients.

Atrophy was observed in the anteromedial prefrontal cortex. This is consistent with previous studies using MRI and FDG-PET^18^. Considering most (but not all) data reported from post-mortem brain tissue, the absence of significant frontal synaptic loss measured in vivo in bvFTD patients is surprising. Tentative explanations may be that FDG-PET and SV2A-PET do not measure the same biological phenomenon and that dementia stages are different in our living participants and in post-mortem studies. The frontal distribution of [^18^F]UCB-H was quite variable in our sample of participants (Fig. 5). At a macroscopic level, elevated connectivity was previously reported in a dorsal portion of the medial prefrontal cortex and in the frontal pole using resting fMRI in bvFTD patients^33,34^. At a microscopic level, SV2A-PET is considered as a proxy of synaptic density measurement, but it precisely targets one protein of synaptic vesicles. Interestingly, the number of vesicles per synapse was shown to be increased in postmortem brain sections from bvFTD patients with PGRN haploinsufficiency, relative to controls^35^. Why this would occur in frontal cortex and not in the anterior parahippocampal region is unclear. In a complementary analysis, we looked for a negative relationship between disease duration and the distribution volume of [^18^F]UCB-H, and there was no significant correlation that would have provided argument for an early increase of synaptic vesicles in the frontal lobe of our patients. Moreover, we did not observe PGRN mutation in our three patients who accepted genetic analyses, while loss of synaptic expression measured in the frontal cortex of postmortem brains of FTLD patients with SNAP-25 immunochemistry was reported to be influenced by different genetic factors^11^.

Our correlation analysis suggests that brain atrophy on MRI and synaptic loss on PET-SV2A reflect different but correlated pathological processes. Synaptic loss has been studied independently from neuronal loss in FTLD^36^. Staging of brain pathology was based on atrophy^37^ and on pathological protein deposits (mainly TDP43 or tau)^28,38^. Atrophy staging was related to neuron loss and gliosis, and not to synaptopathy^39^. Accordingly, information is currently lacking on the sequence of pathological involvement of neurons such as Von Economo ones, particularly affected in bvFTD^40^, and their synaptic projections.

Our second result was a significant correlation between greater anosognosia for the clinical symptoms in bvFTD patients and lower synaptic density in the caudate nucleus and the frontal pole. It is well recognized that beyond behavioral symptoms, patients with bvFTD often exhibit early and severe overestimation of cognitive abilities. In previous studies, anosognosia for cognitive impairment was correlated to atrophy in the subgenual cingulate cortex^41^. The correlation between anosognosia for behavioral symptoms in bvFTD and grey matter atrophy did not show any region surviving correction for multiple comparisons^42^, while negative correlation was observed with glucose uptake in the superior part of the temporal pole^43^. In studies where different patient groups were combined (including patients with bvFTD), impaired self-awareness was already correlated to medial prefrontal cortex atrophy^44^.

Functional connectivity between the head of the caudate nucleus and the medial prefrontal cortex was previously reported^45^, and recent connectomic analysis of the caudate nucleus demonstrated widespread connections with frontal associative cortical regions, comprising areas involved in self-awareness^46^. Our results are adding the head of the caudate nucleus in a network subserving self-awareness, where the caudate nucleus and the pregenual ventromedial prefrontal cortex would participate in self reference processing^47^, associating a value for the self to cognitive or behavioral information^48^. Disconnection in such a network was previously reported in bvFTD^33^, glucose metabolism was already shown to be decreased in caudate and medial frontal regions in bvFTD^49^, and our correlation analysis demonstrates this network’s involvement in impaired awareness of the patients.

There are several limitations to the study. The sample of bvFTD patients is limited and it comprises participants with a clinical diagnosis at a very mild to moderate stage, without genetic or pathological confirmation. Since plasma neuronal-derived exosomes levels of synaptotagmin and synaptophysin were shown to be decreased before dementia is diagnosed in patients with bvFTD^50^, participants at very early stages could be studied in the next future. We discussed trends for significance in our bvFTD sample, and we compared the results to data previously obtained in AD participants, but we did not observe significant between group differences. The PET methodology using carotid artery activity as an input function for kinetic modelling was previously described^21,51^. It certainly deserves further validation and currently it can only provide values of total distribution volume and not values of specific binding potential. A recent article on [^11^C]UCB-J PET acquisitions in AD showed that cerebellum could be used for calculating distribution volume ratio^52^. However, cerebellar changes have been reported in bvFTD^53^. Finally we used a validated measure of anosognosia that is certainly applicable to bvFTD but it does not allow to disentangle cognitive from emotional processes that are subserved by different brain regions.

In conclusion, loss of synaptic projections in our sample of bvFTD patients tended to be predominant in the right anterior parahippocampal gyrus, probably related to previously reported lesions of the uncinate fasciculus. The right anterior parahippocampal gyrus is part of the limbic network and the perceptive social brain, that are target functional networks in the disease. A validated measure of anosognosia, a characteristic but variable symptom in bvFTD, was related to synaptic density in two interconnected regions, caudate nucleus and anteromedial prefrontal cortex. The regions are involved in self-referential processes, that are important in developing awareness of clinical impairment. Further studies should localize decrease of synaptic projections in prodromal bvFTD and use other techniques, such as MRI with neurite orientation dispersion and density imaging (NODDI) to further explore the precise distribution of the synaptopathy.

## Material and methods

### Participants

Three groups of 12 older participants matched for age, sex and education were included in the study (Table 1). The first group comprised 12 participants (four women and eight men) with probable bvFTD^18^, followed in memory clinic. The age range was wide, from 63 to 83 years old. Disease duration was 5.2 ± 3.4 years. Apathy was observed in 11 participants, stereotyped behavior and anosognosia in eight, loss of empathy and dietary changes in five and disinhibition in three. Clinical Dementia Rating was very mild to moderate^54^. None had visuospatial deficits, but most complained of memory (n = 11) and executive (n = 8) difficulties. Familial history of neurodegenerative disorder was reported in seven participants. Visual examination of clinical [^18^F]FDG-PET showed frontal and/or temporal hypometabolism. Delay between [^18^F]FDG-PET and [^18^F]UCB-H-PET was highly variable, with a mean of 22.6 ± 23.5 (range 1–72) months. Accordingly, [^18^F]FDG-PET acquired on different clinical machines could not be compared to SV2A-PET data, and they were not analyzed as experimental data. One participant was amyloid-PET negative. Patients were offered genetic testing (tau, GRN, C9orf72), but only three accepted.

The second group comprised 12 amyloid-positive patients (five women and seven men) with very mild to mild probable Alzheimer’s disease (AD). The diagnosis was based on current NIA-AA criteria^55^. Disease duration was 6.2 ± 3.9 years. As part of the initial diagnostic process, [^18^F]FDG-PET was used as a biomarker of neurodegeneration in seven patients. Amyloid-β positivity was demonstrated in all patients by qualitative visual inspection of [^18^F]Flutemetamol-PET.

The third group was composed of 12 cognitively healthy older participants (CTRL, five women and seven men) with Mini Mental State Examination (MMSE) score greater than 27/30^56^. In this control group, amyloid-negativity was confirmed in four participants using [^18^F]Flutemetamol-PET, while there was no biomarker-related information for the others. The AD and the control groups were selected from previously published populations^21^. Written informed consent was obtained from all participants. The study was conducted in accordance with the declaration of Helsinki. The study (2014/21) was approved by the Ethics Committee of our University (Comité d'Ethique Hospitalo-Facultaire Universitaire de Liège-707).

### Neuropsychological assessment

Global cognition was assessed with the MMSE. As diagnosis was done at the Memory Clinic, we focused our investigation on few tests and questionnaires guided by theoretical interest in the functions of the frontal and temporal brain areas. All participants performed a neuropsychological test known to be sensitive to early visual recognition memory deficits related to medial temporal lobe dysfunction, DMS48^57^. Phonological verbal fluency was measured with letter P during 2 min. Everyday life cognitive functioning and self-awareness were assessed by means of informant-based reports and participants’ reports with the Anosognosia Questionnaire for Dementia (AQ-D) that probes cognitive and behavioral changes^58^, and the Memory Awareness Rating scale-Memory Functioning Scale (MARS-FS) that addresses specifically memory impairment^59^.

### Cerebral image acquisition

Dynamic PET acquisitions were performed using a Siemens/CTI (Knoxville, TN) ECAT EXACT HR + PET scanner. 155.93 ± 9.23 MBq of [^18^F]UCB-H^60^ were injected as an intravenous bolus (injected mass, 0.25 ± 0.22 μg). A thermoplastic mask was used to restrain motion during the acquisition. The timeframe of the dynamic PET was 6 × 10, 8 × 30, 5 × 120, and 17 × 300 s (total = 100 min). All PET images were reconstructed using a filtered backprojection algorithm (Hann filter, 4.9 mm FWHM) and corrected for attenuation using a 10 min transmission scan with Germanium-68 sources, dead time, random events, and scatter using standard software (ECAT 7.1, Siemens/CTI, Knoxville, TN). With these acquisition and reconstruction settings, the transaxial image resolution is 6.5 mm in the center of the axial field of view (FOV) and the voxel size 2.57 × 2.57 × 2.43 mm^3^. Blood samples were collected via a catheter inserted in an arm vein in ten subjects (two bvFTD, five AD, three CTRL) 3, 5, 15, 45, 60, and 90 min post injection in order to determine the plasmatic parent fraction with high performance liquid chromatography. A mean unchanged plasma fraction was calculated for each group and used for modelling^51^. Time by group interaction of plasma fraction was not significant. In addition, participants underwent a whole-brain quantitative MRI protocol on a 3 T Siemens (Erlangen, Germany) Prisma scanner. Multiparameter mapping was based on multi-echo 3D fast low angle shot at 1 mm isotropic resolution^61^. This included three datasets with T1, proton density (PD), and magnetization transfer (MT)–weighted contrasts imposed by the choice of the flip angle (FA = 6° for PD & MT, 21° for T1) and the application of an additional off-resonance Gaussian-shaped RF pulse for the MT-weighted acquisition.

### Image processing

MRI multiparameter maps were processed with the Voxel-Based Quantification (VBQ) toolbox^61^ and SPM12 (Wellcome Trust Centre for Neuroimaging, London, UK) to obtain notably a quantitative MT map as well as segmented images (grey matter, white matter, CSF, “other”), normalized to the standard MNI space using unified segmentation^62^. Modulated normalized grey matter images, resized to 2 mm isotropic voxel size to approximate to PET images, were smoothed with an isotropic Gaussian kernel of 8 mm of full-width at half maximum and analyzed with voxel-based morphometry to identify atrophied regions in the patient groups. The modulated normalized grey matter images across the study sample were used to create a grey matter mask for the statistical analyses. The PET dynamic images were coregistered to the participant’s structural MT image (taking the sum of frames between 2 and 30 min as source image). Then, because of the low resolution of the PET scan and because of our main interest in small structures like the hippocampus, the images were corrected for partial volume effect (PVE) using the “iterative Yang” voxel-wise method implemented in the PETPVC toolbox^63^, with grey matter, white matter, CSF and “other” as ROI masks.

The ubiquitous distribution of [^18^F]UCB-H in the brain did not allow the identification of a “reference region” (with all its necessary characteristics) for a simplified modelling of the radiotracer distribution. Moreover, the need of an arterial input function (AIF) is a heavy discomfort for the patient. An alternative method using image-derived input function was shown to be comparable to that using the AIF for [^18^F]UCB-H PET imaging, even if the coefficient of variation for measurements was slightly increased^51^. So, the input function was derived from dynamic PET images. Briefly, the method extracts time series of radiotracer activity in the carotid arteries^64^. The identification of voxels belonging to the carotids is based on the computation of the Pearson product-moment correlation coefficient between a “seeding region” and voxels in a mask containing the carotid. As, radioactivity is mainly localized in the vessels during the first 2 min, inducing a large spill-out effect, the signal was corrected for this spill-out effect using the geometric transfer matrix approach^65^. For each group, the extracted signal was then corrected using the corresponding mean unchanged plasma fraction to obtain the input function used for modelling. Kinetic modelling using PVE-corrected dynamic PET data and image-derived input function was done with PMOD (Version 3.7, PMOD Technologies, Zurich, Switzerland). Logan graphical analysis was used to calculate the distribution volume (Vt) map of [^18^F]UCB-H in the brain. The t* for Logan analysis was 25 min. Finally, the Vt map was normalized into the MNI space using transformation parameters obtained during structural MRI spatial normalization (2 mm isotropic voxel size).

### Statistical analyses

For SPM12 statistical analyses, the normalized PVE-corrected Vt maps were smoothed with an isotropic Gaussian kernel of 8 mm of full-width at half maximum. These Vt maps for the three groups were entered in a factorial design matrix where two-sample t tests contrasted images 2 × 2. Parameters were estimated according to the general linear model at each voxel, using a grey matter mask. Patient-related regional synaptic loss was tested by a linear contrast (patients–controls) with a statistical threshold of p < 0.05 with a family-wise (FWE) correction for multiple comparisons at the voxel level (no minimal cluster size). Moreover, SPM12 multiple regression models allowed to test for correlation between regional [^18^F]UCB-H distribution volume and each cognitive measure (MMSE, DMS48 proportion of correct responses, verbal fluency score, discrepancy scores between patients’ and informants’ assessments regarding cognitive and behavioral functioning, indicative of anosognosia) with a statistical threshold of p < 0.05 FWE-corrected at the voxel level (no minimal cluster size). VBM was performed on MT images using SPM12 to compare gray matter images between groups.

## Supplementary Information

Supplementary Information.

## Data Availability

The data are available upon reasonable request.
