# Supplementary material for: In vivo exploration of synaptic projections in frontotemporal dementia
Source: Sci Rep. 2021 Aug 9;11:16092. doi: 10.1038/s41598-021-95499-1 (PMC8352914; doi:10.1038/s41598-021-95499-1)

**In vivo exploration of synaptic projections in frontotemporal dementia**

Eric Salmon^1*^, Mohamed Ali Bahri^1^, Alain Plenevaux^1^, Guillaume Becker^1^, Alain Seret^1^, Emma Delhaye^1^, Christian Degueldre^1^, Evelyne Balteau^1^, Christian Lemaire^1^, André Luxen^1^ & Christine Bastin^1^

^1^GIGA Cyclotron Research Centre, University of Liège, B30 Sart Tilman, 4000 Liège, Belgium

*Corresponding author

**Supplemental material**

Supplemental Table 1

Demographic characteristics of participants for resting-state fMRI data

|  | Healthy older participants  n = 27 |
| --- | --- |
| Gender (F/M) | 14/13 |
| Age (years) | 73.9 (6.3) |
| Education (years) | 14.0 (3.8) |
| DRS | 138.6 (3.5) |

Values are expressed as mean (SD). DRS = Dementia Rating Scale.

Supplemental Figure 1. Connectivity of the parahippocampal seed region in a control population, superimposed on an inflated brain MRI template.


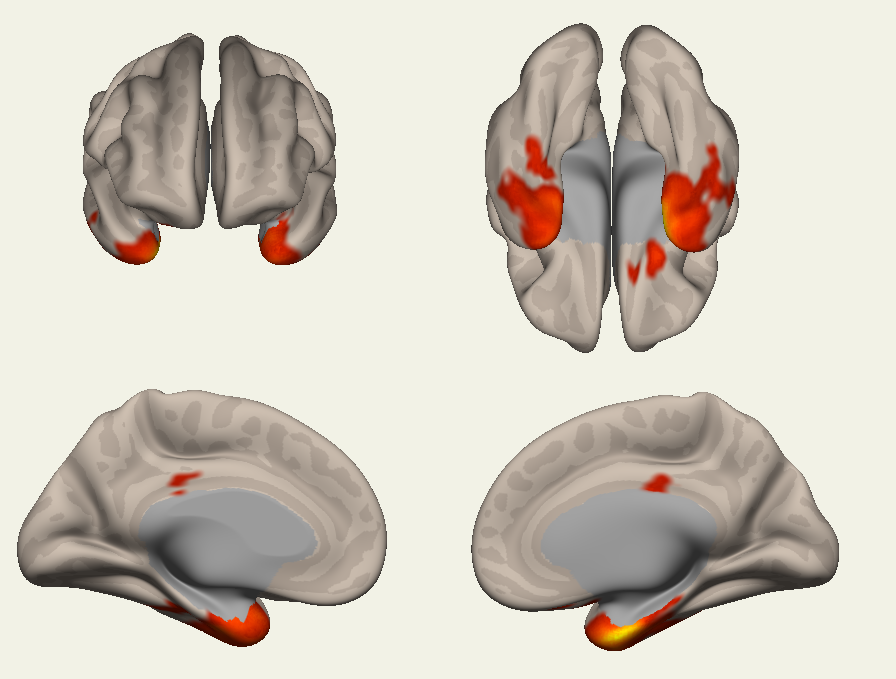


Supplemental Figure 2. Connectivity of the caudate seed region in a control population, superimposed on an inflated brain MRI template.


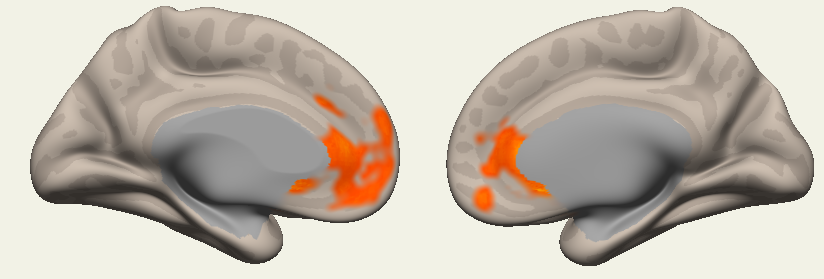

Supplement: Supplementary file 1 — Supplementary Information. [file 41598_2021_95499_MOESM1_ESM.docx]
